# Finite Element Analysis of a 3D-Printed Acetabular Prosthesis for an Acetabular Defect According to the Paprosky Classification

**DOI:** 10.3390/ma18061295

**Published:** 2025-03-15

**Authors:** Mario Ceddia, Giuseppe Solarino, Alessandro Pulcrano, Antonella Benedetto, Bartolomeo Trentadue

**Affiliations:** 1 Department of Mechanics, Mathematics and Management, Politecnico di Bari University, 70125 Bari, Italy; bartolomeo.trentadue@poliba.it; 2 Department of Translational Biomedicine and Neuroscience, University of Bari, 70125 Bari, Italy; giuseppe.solarino@uniba.it (G.S.); alepulcrano@gmail.com (A.P.); benedettoantonella95@gmail.com (A.B.)

**Keywords:** customized acetabular prosthesis, 3D printing, type III acetabular defects, FEA

## Abstract

The treatment of Paprosky Type III acetabular defects is a significant challenge in orthopedic surgery, as standard components often do not fit properly. This study aims to evaluate the biomechanical efficacy of a custom 3D-printed PEEK acetabular prosthesis compared to a conventional titanium implant. A 3D model of the pelvis was created using a computed tomography scanner and a custom-made acetabular implant was designed. Finite element analysis (FEA) was performed using Ansys Workbench to evaluate the stress and strain distribution of two materials on the pelvic bone. The results showed that the titanium prosthesis model had less strain transmitted to the bone, while the PEEK model had better stress transmission and bone stimulation. The use of custom implants reduced the risk of stress shielding, potentially improving long-term bone health. Three-dimensional-printed acetabular prostheses therefore offer significant advantages over traditional implants, suggesting improved implant stability and reduced failure rates.

## 1. Introduction

Each year, more than 600,000 total hip arthroplasties (THAs) are performed in Europe, with approximately 1.4 million procedures conducted worldwide. This number is projected to rise significantly by 2030 due to the aging population and the increasing trend of operating on younger patients [1,2,3]. The Paprosky classification system is widely used to categorize acetabular bone loss in THA revisions, with type III defects being particularly challenging from a reconstructive perspective [4,5,6]. Effective management of these defects requires meticulous surgical planning, as surgeons aim to restore the anatomical center of the hip and maintain structural continuity between the ischium and ilium [7]. This underscores the need for advanced solutions to achieve stable fixation and optimal hip function restoration, as conventional acetabular implants may be insufficient for such complex cases [8,9,10]. Despite technological advancements, failure rates remain high, and clinical outcomes are highly variable. Reported revision rates for the acetabular component range from 20% to 36% within the first ten years [11]. Moreover, successful acetabular revision depends on adequate bone support and an implant design that facilitates biological integration and healing [12]. Several reconstruction methods are available, with the optimal approach depending on the severity of bone loss. Historically, anti-protrusion cages have been the preferred choice for managing these defects; however, their use has been associated with high failure rates [13]. One major limitation of non-custom acetabular components in cases of extensive acetabular defects is their standard sizing, which may not adequately conform to the unique geometry of the defect [13,14].

The introduction of custom implants has addressed these concerns by enhancing implant fit and stability. In recent years, the application of 3D printing technology in medicine has expanded, leading to increased utilization of 3D-printed implants in orthopedic surgery [15,16,17]. Furthermore, recent studies have reported satisfactory mid- to long-term outcomes with 3D-printed acetabular prostheses [18,19,20]. For instance, a study by Taunton et al. [21] evaluated 57 patients with pelvic discontinuities over a follow-up period ranging from 24 to 215 months. The results demonstrated an overall implant survival rate of 95%, with an average Harris Hip Score (HHS) of 74.8, indicating significant functional improvement. Similarly, Berasi et al. [22] reported the use of 28 custom-made triflange implants in 26 patients undergoing acetabular revision surgery. While two patients experienced failure due to infection, no cases of implant loosening were observed. The average HHS improved from 42 to 65. Additionally, DeBoer et al. [23] investigated 30 custom implants in 28 patients with pelvic discontinuities over a mean follow-up period of 10 years, reporting no cases of mechanical failure. Christie et al. [24] reported a dislocation rate of 7.8% in a series of 78 patients, with no cases of mechanical failure. Currently, entire acetabular implants are manufactured using additive manufacturing technology, in which successive layers of titanium are fused via electron beam melting to ensure structural integrity [25].

One of the primary concerns with 3D-printed cast metal prostheses is the uncertainty regarding their long-term fatigue failure risk compared to that of conventionally CNC-machined prostheses [26]. However, preliminary reports on custom 3D-printed metal implants for total hip arthroplasty have been promising. Wyatt et al. [27] conducted a systematic review of seven studies involving 243 custom-made acetabular components for Paprosky type III defects and pelvic discontinuities, demonstrating a low probability of mechanical failure. The accuracy of prosthetic fit is a critical determinant of surgical success. Oversized implants can result in suboptimal fit, local stress concentrations, and instability, whereas triflange implants are often designed to be slightly undersized. This undersizing can lead to inadequate bone-to-implant contact in the central acetabulum, with load transfer occurring primarily through the flanges. As a result, stress shielding may develop in the underlying trabecular bone, reducing mechanical stimulation and potentially leading to progressive bone resorption, implant loosening, and compromised pelvic stability.

Modern additive manufacturing technologies, such as selective laser melting (SLM), enable the production of patient-specific implants using ductile metals such as commercially pure titanium. A study by Magré et al. [28] demonstrated that deformable titanium acetabular implants yielded promising results in terms of primary stability and load transfer. This biomechanical advantage has the potential to stimulate bone growth, thereby enhancing secondary fixation. However, the use of materials with stiffness significantly exceeding that of bone can alter physiological stress distribution. Inadequate stress transmission may result in regions where mechanical stimulation is insufficient to maintain bone homeostasis, leading to localized bone resorption due to stress shielding [29,30,31,32,33,34].

To address this issue, polymeric materials such as polyetheretherketone (PEEK) have been proposed as an alternative for use in total hip arthroplasty (THA) [35,36]. PEEK exhibits a significantly lower Young’s modulus (approximately 3.6 GPa) compared to titanium (110 GPa), thereby reducing implant stiffness and allowing for a more physiological load distribution. In contemporary orthopedic biomechanics, computational modeling approaches, particularly finite element (FE) analysis, have been extensively employed to evaluate stress and strain distributions in hip endoprostheses. The FE method has proven highly effective in reducing the cost and duration of biomechanical studies, making it a fundamental tool for understanding the global biomechanical behavior of implant systems. For instance, a finite element analysis (FEA) study by Ceddia et al. [37] demonstrated that a carbon fiber-reinforced PEEK femoral stem resulted in a more homogeneous stress distribution within the bone tissue compared to a Ti-6Al-4V stem, suggesting a significant reduction in stress shielding. Similarly, another FEA study [38] evaluated the effects of titanium and PEEK in spinal fixation prostheses, revealing that PEEK implants facilitated a more even stress distribution across adjacent vertebrae while providing greater mobility compared to rigid titanium fixation. A comparative analysis by Abdal et al. [39] investigated acetabular cups fabricated from different materials, specifically 30% carbon fiber-reinforced polyetherketone (30 CF/PEEK) versus ultra-high-molecular-weight polyethylene (UHMWPE). Their findings indicated that CF/PEEK acetabular cups, particularly when combined with alumina, exhibited superior mechanical properties compared to UHMWPE, demonstrating higher strength and reduced deformation under load, which may enhance their suitability for clinical applications.

While some FEA studies have analyzed stress distribution in custom-made titanium prostheses, limited research has focused on the biomechanical effects of stress transfer to the pelvic bone when using a PEEK prosthesis. Therefore, the objective of this study is to evaluate the biomechanical performance of titanium and PEEK in the design of a prosthesis for the reconstruction of a Paprosky type III acetabular defect. The primary hypothesis is that PEEK may offer biomechanical advantages over titanium by enhancing bone stimulation while maintaining sufficient fatigue resistance.

## 2. Materials and Methods

### 2.1. Modeling

**Figure 1:**
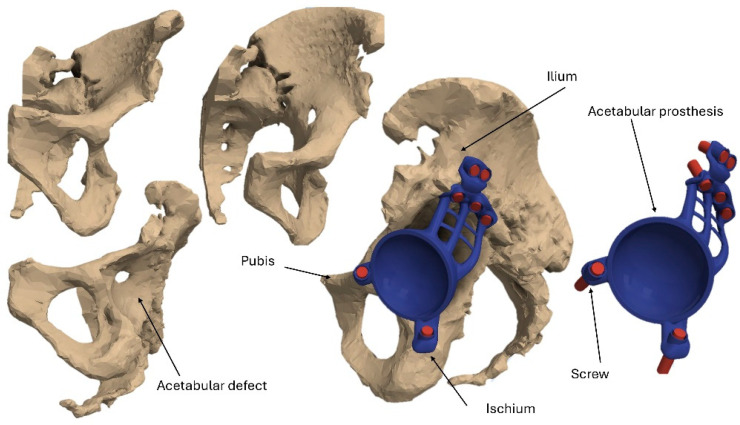
Three-dimensional models of anatomical structures and acetabular prosthesis.

A 3D reconstruction of the pelvis with a Paprosky type III acetabular defect was performed in a young patient weighing 50 kg (Figure 1) [40]. The 3D pelvic model was generated using a new-generation high-resolution (0.5 mm per voxel size) multi-slice computed tomography (CT) scan. This model was then imported into Autodesk Inventor 2024, serving as the foundation for the digital design of the acetabular prosthesis. The design of the prosthesis comprises several key components: the acetabular cup, the iliac flange, the pubic flange, and the ischial flange. To secure the prosthesis to the bone, seven screws, each with a diameter of 6.7 mm and a length of 37.5 mm, were incorporated. These screws were modeled as cylindrical elements, as the primary objective of this study is to evaluate the stress distribution and load transfer between two different prosthesis materials—PEEK and titanium.

**Figure 2:**
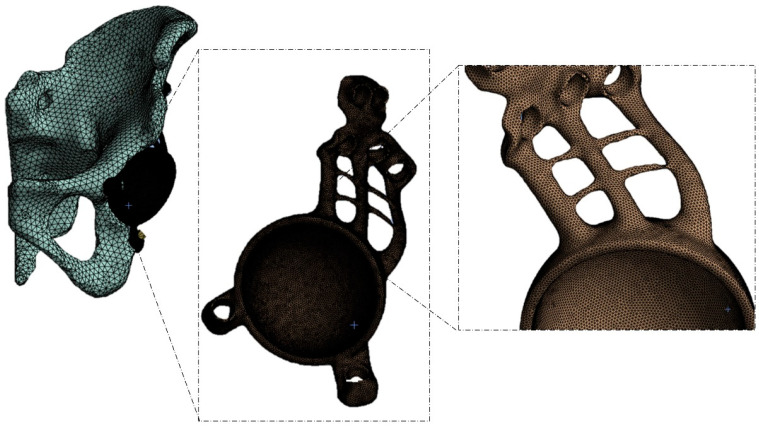
Discretized model with SOLID 187 tetrahedral elements.

To perform the finite element analysis, the converted solid model in (.step) format was imported into Ansys Workbench R2023. The mesh was generated by selecting the automatic mesh option with an average element size of 1 mm, also in accordance with previous studies found in the literature [41,42,43,44] (Figure 2). Due to the complexity of the geometry, SOLID 187 tetrahedral elements were used. This element is capable of accurately modeling the deformation of isotropic or anisotropic materials with complex geometries [45]. With these settings, a total of 173,844 elements and 303,315 nodes were obtained.

### 2.2. Materials

It is essential to pay particular attention to the physical and mechanical properties of bone tissue. The pelvis is composed of low-density cancellous bone and a thin, dense cortical layer that transfers most of the load, while the cancellous bone acts as a support to prevent the cortical bone from collapsing [45,46]. The mechanical properties of bone can change with age and other causes [45,46,47]. Bone tissue is anisotropic, and the distribution of Young’s modulus is linearly dependent on bulk density, as shown in Equation (1) [48].

(1) $$E\left(\mathrm{GPa}\right)=3.79^{0.06}\cdot \rho_{app}^{3}$$

For the pelvis, however, the variations in the Young’s modulus are negligible in the compact, spongy tissue. Using computed tomography, it is possible to calculate the distribution of Hounsfield units (CTs) and relate it to the bulk density $\rho_{app}$ using the following Equation (2) [48].

(2) $$\rho_{app}\left(\frac{g}{{\mathrm{cm}}^{3}}\right)=0.0007918*HU+0.4718988$$

as the CT data available in the literature did not provide precise information with regard to the definition of bulk density, an average value of 0.425 g/cm^3^ was assigned based on the study by Taddei et al. [49]. Furthermore, by interpolation of the measured Hounsfield unit (HU) values, a lower threshold of 500 MPa and an upper threshold of 20,000 MPa were established to adequately represent the stiffness limits of cancellous and cortical bone, respectively. These results are presented in Table 1 [30,49,50,51,52]. However, many finite element analysis (FEA) studies on the pelvis assume the pelvic bone to be isotropic and linearly elastic [30,50,51,52]. The acetabular prosthesis and screws were initially modeled using Ti-6Al-4V. Subsequently, polyetheretherketone (PEEK) was considered as an alternative material for comparative analysis.

### 2.3. Loading and Boundary Conditions

**Figure 3:**
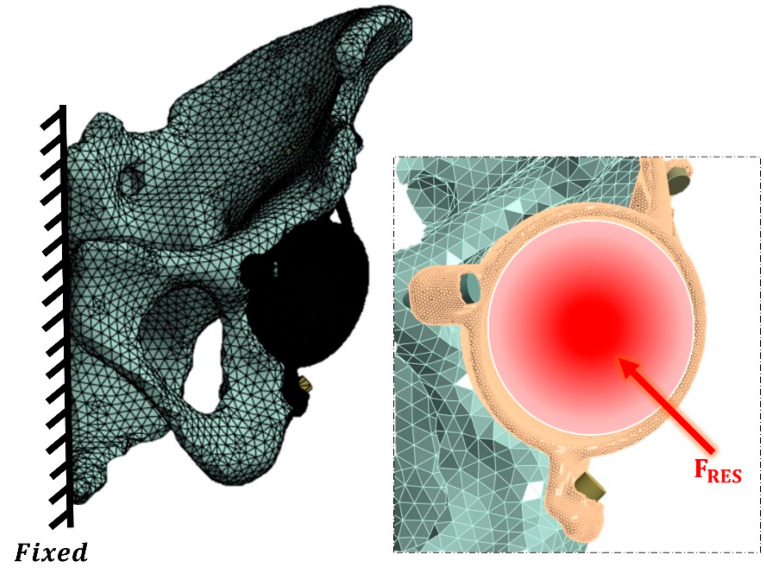
Constraint and load conditions.

The boundary conditions and contact interactions were defined using Ansys Workbench software R2024. A frictional contact interface was assigned between the implant and the pelvic bone, with a friction coefficient of 0.2 [52]. Screw heads were connected to the implant using a frictionless linear contact model with separation allowed. A load of 784.8 N, representing physiological walking load conditions, was applied through a reference point on the acetabular prosthesis (Figure 3). This load was decomposed into its components along the x, y, and z axes: Fx = 502.3 N, Fy = −78.5 N, and Fz = 2048.3 N. Additionally, the model was constrained in all degrees of freedom by applying boundary conditions to all nodes on the hemipelvis half-surface (Figure 3) [53]. To account for initial fixation, a preload of 50 N was applied to the screws [52,53].

The corresponding bone responses, based on the studies of Biewener and Frost [54,55], were used to analyze the stresses and strains. These values in strain (μm/m) are given below:

- <400 μm/m: Atrophy.
- 400–3000 μm/m: Bone preservation and formation.
- 3000–20,000 μm/m: Plastic deformation.
- 20,000 μm/m: Fracture.

## 3. Results

### 3.1. Stress Distribution

**Figure 4:**
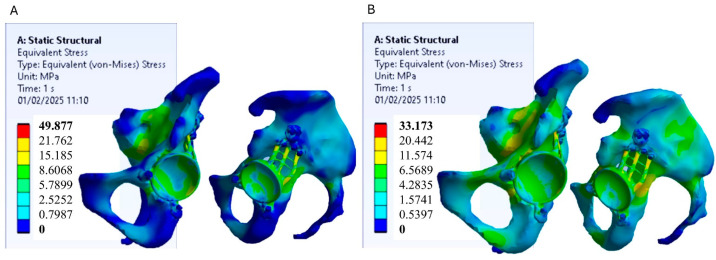
Results of the von Mises stress on the pelvic bone and the acetabular prosthesis. (**A**) model with titanium prosthesis; (**B**) model with PEEK prosthesis.

Finite element analysis (FEA) conducted using Ansys Workbench enabled the assessment of stress distribution and deformation in two acetabular prosthesis models, one made of Ti-6Al-4V titanium alloy and the other of polyetheretherketone (PEEK). The analysis was based on the von Mises stress criterion, which is a widely adopted method for predicting material failure under complex loading conditions. This criterion facilitates the comparison of actual stress values with material strength limits obtained from uniaxial tensile tests, providing a robust framework for result interpretation. Figure 4 shows the stress distribution models for the two prosthesis models.

**Figure 5:**
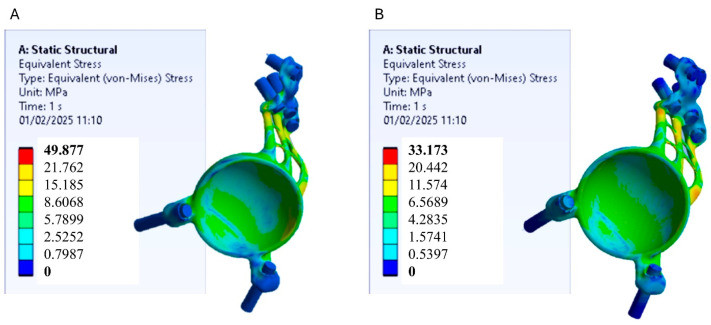
Von Mises stress results in the acetabular prosthesis. (**A**) Model with titanium prosthesis; (**B**) model with PEEK prosthesis.

In the titanium prosthesis model, the highest stress concentrations are primarily located within the acetabular prosthesis itself. This is attributed to the significantly greater stiffness of titanium compared to the surrounding pelvic bone, causing the prosthesis to absorb most of the applied loads while shielding the underlying bone from stress. However, from the perspective of mechanical strength, these stress levels are not critical, as they remain well below the yield strength of titanium, as illustrated in Figure 5. Conversely, the PEEK prosthesis model exhibits a more uniform stress distribution, with loads spread over a larger area of the pelvic bone. Due to its lower stiffness relative to titanium, PEEK facilitates improved stress transfer to the surrounding bone, promoting a more physiological load distribution. Also in this case, the stress values in the PEEK prosthesis remain below its yield strength, confirming it is mechanically safe under the applied loading conditions.

### 3.2. Strain Distribution on the Bone

**Figure 6:**
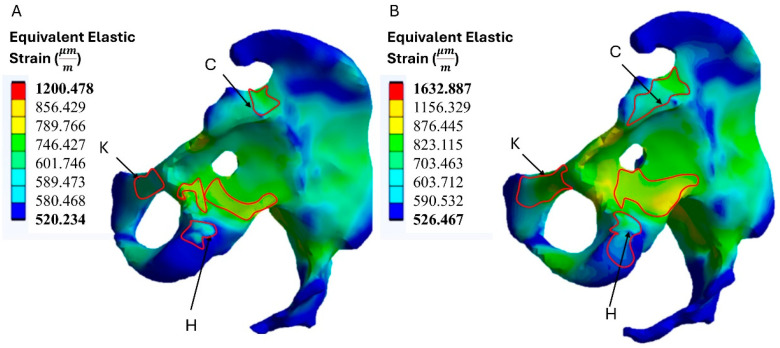
Pelvic bone loading results. (**A**) Model with titanium prosthesis; (**B**) model with PEEK prosthesis.

The resulting deformations in the hemi-pelvis were influenced by the material of the acetabular prosthesis. In the model with a titanium prosthesis, the strains transmitted to the bone tissue were lower than in the model with a PEEK prosthesis. The maximum strain values obtained were 1200.478 μm/m. The reduced strain is due to the greater stiffness of titanium, which limits the transfer of stress to the bone. This behavior can be advantageous in terms of reducing the risk of excessive deformation but could also limit the mechanical stimulation of the bone, with possible long-term implications. In the PEEK prosthesis model, the deformations transferred to the bone tissue were greater, with maximum strain values of 1632.887 μm/m (Figure 6). The higher strain can be attributed to the lower stiffness of PEEK, which allows better stress transfer to the bone and may promote better mechanical stimulation of the bone, reducing the risk of bone atrophy.

The highest strain variation occurred in the acetabular fossa, where strain increased with the use of PEEK compared to titanium. Slight variations were observed in the ischial and pubic regions. Table 2 shows the strain values in the areas where the prosthesis comes into contact with the bone. These areas correspond to those where holes are drilled to allow insertion of the screws and fixation of the prosthesis to the pelvic bone.

Both models generate strains in excess of 400 μm/m, indicating that there is no risk of bone atrophy. However, the model with the PEEK prosthesis produces higher levels of strain than the model with the titanium prosthesis, particularly in the flange attachment areas. This trend suggests that the bone is stimulated more when loaded by the PEEK prosthesis, which may be beneficial for long-term bone health.

## 4. Discussion

### 4.1. Using 3D Printing in THA

In the revision of total hip arthroplasty (THA), managing extensive acetabular defects can be challenging, especially in the presence of pelvic discontinuity. Several implant options, including jumbo cups, acetabular cages, supplemental plates, and allografts, have been proposed to address these complex defects [56]. However, the survival rates of these techniques vary in the literature. For instance, in a retrospective study conducted by Lee et al. [57] focusing on large uncontained acetabular bone defects, 74 patients were treated with acetabular bone grafts and cemented cups. The study reported implant survival rates of 67% at 15 years and 61% at 20 years. Rogers et al. [58] reported an 86.3% survival rate for 42 cup-cage reconstructions in cases of chronic pelvic discontinuity with an eight-year follow-up. Amenabar et al. [59] published midterm results on cup-cage constructs used in 45 patients, noting a failure rate of 9% (4 out of 45), with a mean follow-up of 6.4 years. Furthermore, the use of standard acetabular implants for the reconstruction of extensive acetabular defects may lead to early aseptic intolerance and the need for additional surgical interventions [60,61]. Recently, advancements in 3D printing technology and additive manufacturing have made it possible to create customized implants, reducing the design times from 2 to 3 months to 3 to 4 weeks. Custom acetabular implants demonstrate a lower risk of these types of complications and revisions [62,63]. Custom triflange acetabular components offer several advantages, including rigid fixation to the remaining host bone (ilium, ischium, and pubis) and a porous coated surface that facilitates biological ingrowth onto the bone, thus providing long-term stability. However, there are notable disadvantages, such as the requirement for advanced imaging, high costs, and the inability to modify the implant intraoperatively. Few published studies have demonstrated the outcomes of these current custom designs. For instance, one study [64] evaluated nine patients and reported an implant survival rate of 88.8% with a median follow-up of 28.8 months, with revision required for only one patient due to failure of bilateral pelvic bone integrity. Additionally, long-term studies indicate that between 20% and 35% of patients with custom implants require subsequent revisions. Another complication is periprosthetic infection (PJI). The incidence of PJI is between 8% and 10%. Broekhuis et al. [65] reported a revision rate of 27% for custom triflange reconstructions. Overall, while high rates of complications and failure are common, most studies indicate promising outcomes with custom components [66,67].

### 4.2. FEM Studies in THA

Recently, finite element (FE) analysis simulation has been extensively utilized in the design of orthopedic implants and preoperative planning, aiding clinicians in gaining a better understanding of the biomechanics of prosthetics [68,69,70,71,72,73,74,75]. Compared to other experimental methods, this approach not only accurately simulates the biomechanical performance of implants but also demonstrates increased efficiency and resource savings. For instance, it can be employed to analyze a wide range of innovative materials, such as advanced metal alloys, composite materials, and bioactive substances used in orthopedics. A finite element study conducted by Abdullah et al. [76] established that CF/PEEK is a promising material for acetabular cups in hip prostheses, significantly outperforming UHMWPE in terms of resilience and performance under load. In another analysis [53], the shells used in hip prostheses, particularly in modular acetabular cup systems made of CFR-PEEK, deformed more than titanium shells, which better stimulated the bone. This deformation helped limit the effects of stress shielding. However, Maslov et al. [53] observed that maximum von Mises stresses were recorded within the screws and along the edges of the holes in the implants, with peaks potentially exceeding the fatigue limits allowed for 3D-printed titanium Ti-6Al-4V under dynamic loading conditions. The use of PEEK has helped to mitigate these issues by better distributing stress across the prosthetic components, thus avoiding stress points that could lead to weaknesses in the mechanical strength of the component. For example, a finite element study conducted by Ceddia et al. [77], evaluating the effect of PEEK in the production of a vertebral fixation prosthesis, observed a more homogeneous distribution of stress and better integration with bone compared to titanium models. Specifically, it was noted that stress values for titanium screws were higher than those for PEEK screws. However, no studies in the literature have analyzed the use of PEEK for creating prostheses used for acetabular defects. The analysis conducted in this study showed that titanium prostheses caused less deformation (strain) in the bone tissue compared to those made of PEEK. The maximum strain values for titanium were recorded at 1200.478 μm/m, while those for PEEK were higher, reaching 1632.887 μm/m. This suggests that titanium prostheses limit the transmission of stress to the bone, potentially preventing excessive deformation; however, this may also reduce the beneficial mechanical stimulation required for bone recovery. Additionally, titanium prostheses tend to concentrate stresses primarily within the prosthesis itself. This behavior reduces the mechanical stimulation of the pelvic bone, increasing the risk of stress shielding, as the underlying bone does not receive sufficient mechanical loading to maintain its mass. In contrast, the PEEK prosthesis demonstrated a more uniform distribution of stress, effectively transmitting loads to the surrounding bone compared to titanium. This suggests that the use of PEEK could promote better mechanical stimulation, which may encourage healthier bone growth around the implant. From a clinical standpoint, the results obtained in this study could guide surgeons to consider alternative materials for acetabular implants, improving clinical outcomes and reducing the risk of long-term complications. Furthermore, the application of 3D printing technology for the creation of custom implants can provide better adaptability to the patient’s anatomy, enhancing implant stability compared to standardized approaches. This study paves the way for further research on innovative materials and 3D printing techniques, leading to future developments in the design and application of orthopedic implants. Despite the promising results of our biomechanical analysis of PEEK acetabular implants compared to titanium implants, this study has several limitations that must be considered.

First, our research is based solely on finite element analysis (FEA) simulations, which, although useful for predicting biomechanical behavior, are not a substitute for direct experimental evidence. Therefore, the lack of preliminary mechanical tests to validate the simulations is a significant limitation. Further experimental testing is needed to confirm the results obtained and to ensure the clinical applicability of PEEK implants.

## 5. Conclusions

This study showed that the use of 3D-printed acetabular prostheses, particularly those made of PEEK, offers significant advantages over traditional titanium implants, especially in situations involving complex acetabular defects according to the Paprosky classification. The greater ability of PEEK to distribute stress more evenly around the implant, reducing the effect of stress shielding, results in a potential improvement in long-term bone health and implant stability. In addition, the results suggest that customized implants can improve anatomical fit, reducing failure rates and the associated complications. Three-dimensional printing technology not only has the potential to reduce the design and production time of implants but could also enable more effective bone protection.

## Figures and Tables

**Table 1:** Mechanical properties of the materials used in the following study.

Material	Young’s Modulus, GPa	Poisson’s Ratio	Yield Stress MPa	Fatigue
Cortical tissue	20	0.3	80–150	The same as the yield stress
Spongy tissue	0.05	0.2	1.4–2.1	
Ti-6Al-4V	113.8	0.3	950	310–610
PEEK	23	0.4	120	80

**Table 2:** Strain results in zones C, K, and H.

Position	Equivalent Elastic Strain ($\frac{\mu m}{m}$)	
	Model A	Model B
C	789.236	867.112
K	745.219	826.983
H	647.521	719.387

## Data Availability

The original contributions presented in this study are included in the article. Further inquiries can be directed to the corresponding author.
